# Is arthrocentesis effective for temporomandibular joint disorders?

**DOI:** 10.1590/acb406425

**Published:** 2025-08-08

**Authors:** Eduardo Grossmann, Sigmar de Mello Rode

**Affiliations:** 1 Universidade Federal do Rio Grande do Sul - Instituto de Biociências - Ciências Morfológicas - Porto Alegre (RS) - Brazil.; 2 Universidade Estadual Paulista - Instituto de Ciência e Tecnologia - São José dos Campos (SP) - Brazil.

**Keywords:** Artrocentesis, Temporomandibular Joint, Temporomandibular Joint Disc, Temporomandibular Joint Disorders, Temporomandibular Joint Dysfunction Syndrome

## Abstract

This review covers the most recent articles on temporomandibular joint arthrocentesis, focusing on its mechanism of action, indications, clinical outcomes and efficacy, complications, comparison with other treatment modalities, and conclusions.

## Introduction

For patients suffering from temporomandibular joint disorders (TMD) who have not found relief with conservative treatments (e.g., physical therapy, or oral appliances, or medications^1^), arthrocentesis offers a minimally invasive alternative. The procedure involves introducing a cannula^2^, a needle or needles^3^ into the temporomandibular joint (TMJ) space to lavage the joint and remove inflammatory mediators, as well as to break up adhesions that can limit movement or cause pain^4^,^5^. Over the years, arthrocentesis has gained popularity as a first-line surgical option^6^ due to its simplicity, minimal invasiveness, and efficacy in treating a wide range of TMD-related symptoms^7^.

## Mechanism of action

Arthrocentesis works by flushing the joint with a saline solution, which helps to reduce intra-articular pressure, eliminate inflammatory substances like cytokines, and mechanically disrupt adhesions that form within the joint space^8^. In certain cases, therapeutic substances such as hyaluronic acid^9^ or injectable platelet-rich fibrin (i-PRF)^10^ are injected into the joint post-lavage to promote lubrication and reduce inflammation, further enhancing the benefits of the procedure.

## Indications for arthrocentesis

Arthrocentesis is most commonly indicated in patients with disc displacement without reduction, in which the disc becomes stuck in a displaced position and does not return to its normal position during jaw movement, resulting in pain, limited opening, or a closed lock^1^,^5^. It is also used in cases of osteoarthritis^11^, rheumatoid and psoriatic arthritis, ankylosing spondylitis of the TMJ, and persistent pain that fails to respond to non-invasive treatments1^2^. TMJ arthrocentesis can help modulate pain, increase mouth opening, and relieve locking^12^. These minimally invasive procedures have few complications^12^ and can be repeated under local anesthesia with minimal downtime^13^,^14^.

## Clinical outcomes and efficacy

A substantial body of clinical research supports the efficacy of arthrocentesis in treating TMD. Most studies report significant reductions in pain and improvements in mandibular range of motion after the procedure. For example, Guarda-Nardini et al.^13^ demonstrated that patients treated with arthrocentesis experienced a marked decrease in joint pain and a significant increase in maximum mouth opening post-procedure, with results sustained over the medium term. Similarly, studies^4^,^5^ have emphasized that arthrocentesis effectively restores joint mobility by releasing the “closed lock” condition, providing both immediate and long-lasting relief.

A research^14^ contributed significantly to the understanding of arthrocentesis in patients with disc displacement without reduction. In some studies^15^-^17^, they found that the procedure not only improved joint mobility but also reduced pain significantly, with long-term results sustained after treatment. These studies support the growing body of evidence that arthrocentesis is a reliable intervention for specific TMD cases, particularly when conservative treatments fail.

Currently, there is no consensus on the best treatment for TMJ disc displacement without reduction (DDWoR). Some authors^18^ conducted a study to determine the best therapy, both conservative and minimally invasive, for this type of displacement. The following were used: occlusal splints, low-level laser therapy, arthrocentesis (Arthro) alone, Arthro and intra-articular injection of platelet-rich plasma (PRP), Arthro and hyaluronic acid (HA), Arthro with exercises, and Arthro plus occlusal splint and manipulative therapy. The variables involved were pain intensity on a visual analogue scale and maximum mouth opening (MMO, mm). The quality of the published studies was very low. In the medium term, Arthro alone or combined with other therapies provided better pain reduction than conservative treatment. Conservative treatment significantly increased MMO in the short term compared to other treatments. These findings suggested that conservative and minimally invasive treatment can be applied simultaneously in patients with DDWoR. New randomized controlled trial studies, with larger sample sizes, are needed to confirm these findings.

## Complications

TMJ arthrocentesis remains a procedure with a minimal number of major complications. If present, they are generally temporary, caused by the anesthetic effect or soft tissue edema created by the extravasation of fluid during the irrigation procedure, and can be treated conservatively. Temporary swelling of the periarticular tissues or external auditory canal, ipsilateral temporary open bite, frontal and orbicularis oculi paresis, preauricular hematoma, and, more rarely, vertigo can be observed during or shortly after the procedure^19^.

## Comparison with other treatment modalities

When compared to more invasive interventions like arthroscopy or open-joint surgery^20^, arthrocentesis offers several advantages. Its minimal invasiveness, short recovery time, and cost-effectiveness make it a viable option for early surgical intervention in TMD management^4^,^5^. Arthroscopy, although more effective in certain cases of complex joint pathology, requires a greater learning curve, presents a greater risk of complications, and requires more specialized equipment^21^,^22^. TMJ arthrotomy is indicated for severe cases, such as ankylosis or tumors^20^. It is reserved for situations in which other treatments fail and is therefore considered a last option for a select group of patients, due to its invasive nature and higher risk profile^23^,^24^.

## Conclusion

Arthrocentesis is an effective, minimally invasive treatment for certain types of TMJ disorders, particularly disc displacement without reduction and inflammatory conditions. It offers significant improvements in pain relief and joint mobility. It serves as an excellent option for patients who have not responded to conservative therapies. Additionally, combining arthrocentesis with other therapies, such as intra-articular injections of hyaluronic acid, i-PRF, or PRP, can increase its effectiveness, further improving long-term results.

## Data Availability

Not applicable
